# Analysis of the Pathogenic Factors and Management of Dry Eye in Ocular Surface Disorders

**DOI:** 10.3390/ijms18081764

**Published:** 2017-08-13

**Authors:** Marcella Nebbioso, Paola Del Regno, Magda Gharbiya, Marta Sacchetti, Rocco Plateroti, Alessandro Lambiase

**Affiliations:** Department of Sense Organs, Policlinico Umberto I, Sapienza University of Rome, Piazzale Aldo Moro 5, 00185 Rome, Italy; paoladelregno@hotmail.it (P.D.R.); magda.gharbiya@uniroma1.it (M.G.); marta.sacchetti@uniroma1.it (M.S.); rocco.plateroti@uniroma1.it (R.P.); alessandro.lambiase@uniroma1.it (A.L.)

**Keywords:** dry eye, lacrimal gland, lipids and lipidomics, Meibomian gland, ocular surface disorders, proteins and proteomics, tear film

## Abstract

The tear film represents the interface between the eye and the environment. The alteration of the delicate balance that regulates the secretion and distribution of the tear film determines the dry eye (DE) syndrome. Despite having a multifactorial origin, the main risk factors are female gender and advanced age. Likewise, morphological changes in several glands and in the chemical composition of their secretions, such as proteins, mucins, lipidics, aqueous tears, and salinity, are highly relevant factors that maintain a steady ocular surface. Another key factor of recurrence and onset of the disease is the presence of local and/or systemic inflammation that involves the ocular surface. DE syndrome is one of the most commonly encountered diseases in clinical practice, and many other causes related to daily life and the increase in average life expectancy will contribute to its onset. This review will consider the disorders of the ocular surface that give rise to such a widespread pathology. At the end, the most recent therapeutic options for the management of DE will be briefly discussed according to the specific underlying pathology.

## 1. Introduction

Dry eye (DE) syndrome, or keratoconjunctivis sicca, is an autoimmune disorder of the ocular surface that affects more than 35% of the population [1], and most frequently the female gender. It consists of qualitative and quantitative alteration of the tear film, whose main function is lubrification, nutrition, optical transparency, cleanliness, and as the main defense to bacterial infections, both corneal and conjunctival.

The Dry Eye Workshop (DEWS) in 2007 defined DE as a multifactorial disease of tears and ocular surface that results in symptoms of discomfort, visual disturbance, and tear film instability with potential damage to the ocular surface, which is accompanied by increased osmolarity of the tear film and inflammation of the ocular surface [2].

**Figure 1:**
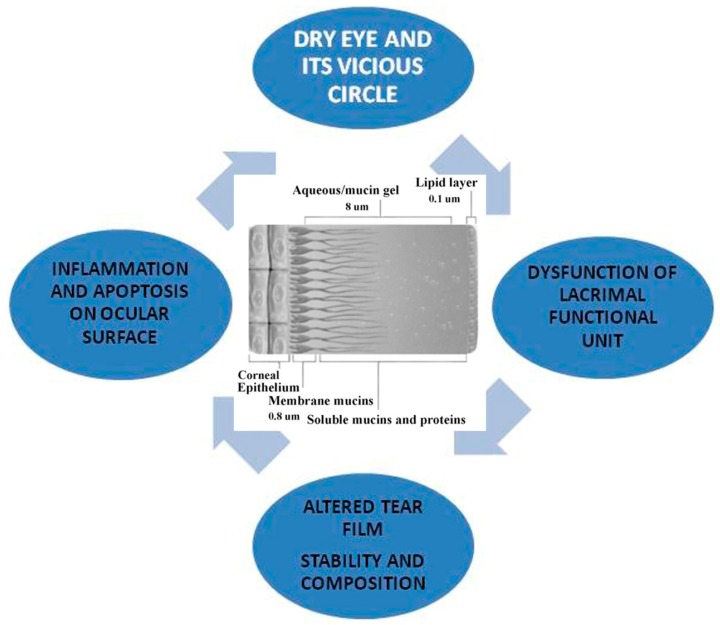
Dry eye and its vicious circle.

The most common symptoms of the disease are burning, foreign body sensation, photophobia, itching, stinging, irritation, redness, blepharospasm, difficulty in opening eyelids on awakening, and, in severe cases, pain and blurred vision. Symptoms are subjective and increase in special environmental conditions, such as wind exposure, dry heat, and low humidity, and in the presence of contact lens (CL), smoking, air conditioning, or heating (Table 1). Each specialized layer of the ocular surface contributes to a component of the tear film (Figure 1). In fact, the lacrimal and accessory glands produce the central nutritive aqueous component; the Meibomian glands (MGs) produce the oily outer lipid that provides tear film stability; and the conjunctival mucipare goblet cells (GCs) produce the inner “surfactant” mucin that primarily improves the wettability of the ocular surface. The eyelids and lashes provide fundamental protection through the blink mechanism, wipe away debris from the ocular surface, and replenish the surface with a fresh layer of tears. Among these various factors, the dysfunction of MGs is one of the most frequent causes found in DE.

In addition, the DEWS recognizes two subgroups of DE based on the etiopathogenesis: evaporative and aqueous deficit. On the one hand, excessive evaporation, caused by the dysfunction of MGs, and mixed forms of DE are found in more than 80% of cases; whereas approximately 10% of patients with DE have only an aqueous deficit [3].

In this review, we have deepened the pathogenetic (Table 2), biochemical, and biophysical mechanisms responsible for DE. We have also reported new therapeutic models in relation to the causes of tear discomfort.

## 2. Methods

A literature study of relevant publications about DE syndrome was performed. Through a computerized search for review, several relevant articles in the PubMed database, published between 2000 and 2017, were identified. Key words used for the search included: “Dry eye” or “Ocular surface disorders” or “Keratoconjunctivitis sicca” or “Sjögren’s syndrome” and “Tear film” and “Lacrimal gland” or “Meibomian gland” or “Lipids and lipidomics” and “Protein and proteomics” and “Treatment” or “Therapy phase II study”. Potentially eligible articles were clinical trials or review articles pertinent to treatment for DE and written in English. Relevant articles were manually searched and reviewed, and data concerning DE were included in the manuscript.

### 2.1. Dysfunction of Meibomian Glands

#### 2.1.1. Primary Dysfunction of Meibomian Glands

The dysfunction of MGs is the most frequent cause of evaporative DE disease. MGs are localized at the level of the upper and lower eyelids, and secrete lipids on the ocular surface, which form the outermost layer of the tear film, with a lubrification function during blinking and a protection function against tear evaporation [4,5]. In 2011, the International Workshop for MGs dysfunction defined the evaporative DE disease “as a chronic, diffuse abnormality of MGs, characterized by terminal duct obstruction and/or qualitative/quantitative changes in the glandular secretion” [6]. The probable cause of this pathology is due to a meibum stasis secondary to the obstruction, secretion, and inflammation of MGs that favors bacterial growth. This could lead to an increase in the release of esterase and lipase by commensal bacteria of the eyelids. As a consequence of this increased enzyme activity, the bacteria change the meibum’s viscosity, leading to further stagnation of meibum within the MGs and generating free fatty acids, which in turn cause inflammation and hyperkeratinization [4,7]. This change of the lipid composition leads to the appearance of foam in the tear film, often visible on the eyelid margin of patients with MG dysfunction [8,9]. The absence of the normal meibum also would reduce the lipid content of the tear film, entering in the vicious circle of DE disease, in which the lipid deficiency of the tear film promotes increased tear evaporation, hyperosmolarity, and inflammation. Furthermore, an infection with commensal bacteria, such as *Staphylococcus aureus*, is one of the causes of chronic blepharitis. Recent studies suggested that a new term be used to describe it as single, chronic disease called “Dry eye blepharitis syndrome” (DEBS) [10]. It is an inflammatory condition of the eyelid margin previously considered synonymous with MGs dysfunction, but, in recent years, has been recognized as a distinct clinical condition that may develop in the later stages of MGs dysfunction, or independently where DE is the ultimate manifestation of other ocular surface disease.

#### 2.1.2. Secondary Dysfunction of Meibomian Glands to Chronic Inflammation

Skin diseases, such as *rosacea*, *seborrheic dermatitis*, or *psoriasis*, etc., also play a role in the pathophysiology of MG dysfunction. Rosacea is a chronic skin disorder that affects the sebaceous glands, especially at the level of the face. It is characterized by erythema, telangiectasia, facial rash, inflammatory papules, and occasionally the hypertrophy of connective tissue [11]. It is more common in people aged between 50 and 60 years [11,12], and the ocular manifestations may be associated with skin manifestations or may appear independently. Although the etiology is uncertain, the latest findings show that microbial pathogenesis and the mechanisms associated with chronic inflammation and autoimmune dysregulation may be involved. The primary defect in ocular rosacea is MG inflammation with dilatation and obstruction of the gland orifices leading to chronic dysfunction. Chronic inflammation can lead to hyperkeratinization of the ductal epithelium, a loss of secretion, and then to blepharitis, meibomitis, chalasia, telangiectasias, and eyelid irregularities [13].

This stimulates the inflammatory cells to release cytokines and activators of matrix metalloproteinases (MMP) leading to ocular surface damage [14,15]. Subsequently, punctate keratopathy and the superficial vascularization of peripheral cornea are also commonly found [12,13].

### 2.2. Dysfunction of Meibomian Glands and Aqueous Deficit: Mixed Forms

#### 2.2.1. Aging and Lacrimal Discomfort

Another major risk factor for DE is age. Modifications of MG structure have been identified with age as an altered localization of the peroxisome proliferator-activated receptor-γ (PPAR-γ), a lipid-activated hormone receptor that regulates lipid synthesis and cell differentiation [16,17] or MG atrophy [18]. Changes in the mucocutaneous junction and glandular atrophy through a loss of meibocyte progenitors have been found in a mouse model [18]. Aging, in fact, leads to a decrease of the acinar diameter and an increase in acinar wall inhomogeneity without significant modifications of the glandular orifice diameter, altering qualitatively the meibum secretion. This is in agreement with the main clinical changes observed during aging, represented by tear break up time (BUT) and Schirmer test scores reduction. Moreover, with aging, we observe the atrophic involution of the glandular unit, and then a progressive dysfunction of the secretive activity. This appears to be in line with the involution of most parts of secretive structures, such as exocrine glands and lymphatics, observed in several tissues of the human body during aging [19].

#### 2.2.2. Aging and Associated Factors

Advanced age is also often associated with several factors.

1. The chronic use of *systemic medications*, such as antidepressants, diuretics, dopamine such as those used for Parkinson's disease, and anti-metabolites commonly used to treat rheumatoid arthritis. Depending on their mechanism of action, these drugs affect lacrimal secretion. In Parkinson’s patients, for example, dopaminergic dysfunction is thought to play a role in decreasing the blink reflex that leads to DE, in addition to a physiological decrease of corneal sensitivity with age that increases the risk of exposure keratopathy. In addition, the decrease in liver and kidney function lengthens the clearance time of systemic drugs.
2. The chronic use of *topical medications* as for glaucoma. β blockers, α-adrenergics, and prostaglandins can reduce tear production. They can cause chronic irritation of the ocular surface that compromises the integrity of the lacrimal glands. It has been highlighted that a reduction of glandular density and area, and increased viscosity of the meibum, can occur. In addition, the presence of preservatives, such as benzalkonium chloride (BAK), can cause tear film instability, GCs loss, conjunctival squamous metaplasia and apoptosis, disruption of the corneal epithelium barrier, and damage to deeper ocular tissues even at low concentrations [20].
3. *Abnormalities in the eyelid positioning*, as laxity, floppy eyelid syndrome, retraction, entropion, ectropion, and lagophthalmos. Horizontal lid laxity is the most common cause of involutional eyelid malposition. Eyelid malposition leads to corneal exposure, poor tear-film distribution, and abnormal tear outflow. As many as 50–70% of patients with this disease develop tear discomfort syndrome [21].
4. *Conjunctivochalasis* is another notable contributor to poor tear outflow, and is characterized by a redundant bulbar conjunctiva interposed between the globe and the eyelid [22]. Pathogenesis of conjunctivochalasis is under investigation; however, elastotic degeneration from cumulative sun exposure and inflammatory degeneration from delayed tear film clearance have been proposed [23]. Once formed, the redundant folds interfere with the inferior tear meniscus, and in some cases cause the occlusion of the inferior punctum.
5. A gradual *reduction in corneal sensitivity* has been shown to occur with increasing age, which predisposes older adults to DE. Roszkowska et al. reported that the mechanical sensitivity of the peripheral cornea decreases gradually throughout life, whereas central corneal sensitivity remains stable until 60, and then decreases sharply subsequently [24].
6. *Oxidative stress*, a counterpart of inflammation, occurs when antioxidants are unable to counteract reactive oxygen species (ROS) that are generated in normal metabolic processes. The production of aggressive oxygen species, such as free radicals and peroxides, leads to DNA damage over time, inducing cell necrosis and the impairment of the regenerative capacity of the corneal epithelial cells. In younger, healthy subjects, low levels of ROS are counteracted by antioxidant enzymes [25].

### 2.3. Aqueous Deficits in Autoimmune Diseases: Sjögren’s Syndrome and Non-Sjögren’s Syndrome Dry Eye

The DEWS divided aqueous deficits in two subclasses: Sjögren’s syndrome (SS) and non-Sjögren’s Syndrome Dry Eye (non-SSDE). SS is a chronic inflammatory disorder characterized by exocrine gland dysfunction. Lymphocytic infiltration of the lacrimal and salivary glands results in the classic sicca complex characterized by DE (keratoconjunctivitis sicca or xeroftalmia) and dry mouth (xerostomia). It can also affect the skin, trachea, vagina, nose, and throat. Patients with DE associated with SS have been found to have elevated levels of interleukin-6 (IL-6) and tumor necrosis factor-α (TNF-α) in their tears [26]. The IL-6 level is associated with disease severity, and was found to correlate with tear film and ocular surface parameters, e.g., tear film, lacrimal meniscus, BUT, Schirmer test, tear clearance, GC density, and keratoepithelioplasty score [26]. Recently, Zhu et al. found that the co-stimulatory molecules Ox40 and Ox40L on peripheral blood mononuclear cells are higher in Sjögren patients than in normal controls, and their levels correlated with clinical outcome and therapeutic response [27]. Even in patients with severe SS, biopsy specimens have revealed that 50% of glandular cells are still present. These results emphasize the importance of immune factors, such as cytokines, MMP, and autoantibodies, in decreasing neurosecretory circuits and inducing glandular dysfunction. The keratoconjunctivitis in SS is classically described as an aqueous tear deficiency. However, in more recent years, this assumption has been challenged. Some studies suggest that pathologic changes induce global tear dysfunction, including alterations in MG function [28]. An analysis of patients affected with SS with aqueous tear deficiency compared to non-SSDE with aqueous tear deficiency demonstrated increased evaporation and also decreased MG secretion, with a deficient lipid layer in the first group [28]. A new laser scanning confocal microscopy approach recently found some differences in the MGs’ features among patients with primary SS, non-SSDE, and MG disease [27,28,29]. In fact, the pattern of inhomogeneity of the MGs’ walls and interstices was markedly higher in all groups of patients than in controls, with features more pronounced in primary SS compared to non-SSDE. This inhomogeneous appearance varies with the level of inflammation, which is significantly higher in eyes with SS, because of the autoimmune pathogenesis of this condition. The presence of confocal signs of inflammation in the absence of dilative morphologic changes supports the presence of an inflammatory/atrophic nonobstructive MG disease in primary SS [30,31]. Diagnosis of SS is generally based on the American-European Consensus Group (2002), and requires at least four out of six criteria, or three out of the four objective criteria. The six criteria include subjective and objective ocular dryness, subjective and objective oral dryness, the presence of Sjogren-specific antibodies A (SSA)/RO and antibodies B (SSB)/LA, and a positive minor salivary gland biopsy [32]. However, in 2012, new classification criteria for SS were approved by the American College of Rheumatology, which require at least two of the following three criteria: positive serum anti-SSA and/or anti-SSB or rheumatoid factor or antinuclear antibody, a total ocular surface staining score >3, and the presence of focal lymphocytic sialadenitis with a focus score >1/4 mm^2^ in labial salivary gland biopsy samples [33]. According to the classification criteria from the European-American collaboration, secondary SS consists of the features of primary SS together with other features of an overt autoimmune connective tissue disease, the most common of which is rheumatoid arthritis. There is a known association between DE syndrome and several systemic diseases, such as SS, rheumatoid arthritis, scleroderma, polymyositis, lymphoma, amyloidosis, hemochromatosis, sarcoidosis, and systemic lupus erythematosus [34]. In particular, rheumatoid arthritis presents the greatest risk of ocular complications, such as scleritis, DE, and peripheral ulcerative keratitis [35].

### 2.4. Other Major Causes of Mixed Forms

#### 2.4.1. Hormonal Changes

DE disease affects women more frequently than men. Women are more likely to experience DE disease during certain periods of significant hormonal alteration, for example, during pregnancy, lactation, oral contraceptive use, and after menopause, suggesting that DE may be related to sex hormones [36,37,38,39]. Receptors for androgens, progesterone, estrogen, and prolactin have been found in several ocular tissues, including the lacrimal gland and MG [36,37,38,39]. Androgens are steroid hormones that are produced by the adrenals (testosterone, androstanediol, dehydroepiandrosterone sulfate (DHEAS), dehydro-epiandrosterone (DHEA), androstenedione (A)), gonads (T), and peripheral tissues (dihydrotestosterone (DHT)). Experimental and human studies have demonstrated that androgen levels are essential for normal lacrimal gland function and structural organization, and that prolactin and estrogen also plays an important role in providing adequate hormone provision for optimal lacrimal production [39,40,41]. In particular, the androgen receptor influences the MGs; this has led to the hypothesis that these glands are under hormonal control. Androgens, in fact, act on epithelial acinar cells that contain receptors for messenger RNA and/or protein receptors for androgens. These cells respond to androgens by binding them to a specific lipid-producing area on the cell surface, which then transcribes specified genes that increase the lipid layer’s distribution over the ocular surface [36,37,38,39]. In particular, Suzuki et al. [38] demonstrated that testosterone stimulates genes related to lipid metabolic pathways and suppresses genes regulating epithelial keratinization.

Moreover, the estrogen produced after menopause derives primarily from the peripheral conversion of adrenal androgens to oestrone in the liver, kidney, brain, and adrenal and peripheral adipose tissue. The absolute levels of oestrogen are influenced by weight, sex, and age [39]. In postmenopause, androgens continue to be produced by the ovarian stromal cells and hilus cells in response to the increased levels of circulating lutein hormone [39]. The data suggest that sex hormone deficiency may not only cause a reduction in the production of aqueous tears, leading to a deficiency, but also a dysfunction in the MG function, resulting in an evaporative DE. In women with complete androgen insensitivity syndrome due to androgen receptor dysfunction, an alteration in the neutral and polar lipid patterns of human MG secretion along with the keratinization of the MG ductal epithelium (i.e., orifice metaplasia) and the lid has been observed [38,39,40,41]. Particularly in these women, there is a significant increase in signs and symptoms of DE. Not surprisingly, a high incidence of evaporative DE disease was found in hyperandrogenic women affected by polycystic ovarian syndrome [40,41]. This allows us to speculate that a perfect balance between androgens and estrogens is necessary to maintain a stable tear film [40,41].

#### 2.4.2. Use of Contact Lenses, Computers, and Interactive Environments

The over use of CL may highly increase the risk of a syndrome of tear film dysfunction. The main observed findings are a decreased basal epithelium cell density, reduced acinar unit diameters, higher glandular orifice diameters, greater secretion reactivity, and greater inhomogeneity of the periglandular interstices. Morphologic changes in the MGs shown by CL wearers were interpreted by the authors as signs of MGs dropout, duct obstruction, and glandular inflammation caused by chronic mechanical CL irritation [12,19,20,30]. In such cases, therefore, it is recommend to use low or medium lenses hydration that would release the liquid, retaining its geometry, high permeability of oxygen, and the use of monodose artificial tears viscous.

Environments characterized by high temperatures, high humidity, wind, smoke, smog, and air conditioning result in a greater rate of tear evaporation. The prolonged use of computer screens, fixation efforts, and the top position of the screen which increases the exposed ocular surface are all factors that may increase tear film evaporation, while the decreased frequency of blinking will reduce tear film stability and efficiency.

#### 2.4.3. Dry Eye and Allergic Diseases

Allergic diseases are among the most common ocular surface disorders, and allergy is one of the most frequent eye problems that occurs in clinical practice. The prevalence of allergic diseases in children between 6 and 14 years varies from 0.3 to 20.5%, and is gradually increasing [42]. This is due to genetic factors, environmental pollution, and exposure to the sun in children. The most common form of allergy is seasonal allergic conjunctivitis (AC), while vernal keratoconjunctivitis (VKC) is considered a rare disease in Europe, most frequently affecting children within the second decade of life. VKC is a serious pediatric multifactorial illness, which compromises the quality of life of children, and can lead to severe ocular symptoms, with giant papillae on the upper tarsal conjunctiva (cobblestoning appearance), and gelatinous infiltrations to the limbus surrounding the cornea, called Horner–Trantas dots. If untreated, ocular surface remodeling leads to corneal ulcers and scars [43]. VKC can be associated with other serious disorders of allergic or autoimmune nature, such as asthma or collagenopathies [43].

There are many examples in the literature that link the dysfunction of the lacrimal film with chronic forms of AC. Pflugfelder et al. [44] suggested that activated T-cells and an increase in inflammatory cytokines, such as epidermal growth factor, IL-1, and IL-8, may damage GCs and the conjunctival epithelium, leading to a reduction of mucin production and subsequent decrease of tear film BUT.

These inflammatory changes cause macroscopic modifications of the MG, represented by glandular atrophy and ductal dilatation. The same morphofunctional alterations were described in seasonal AC, where we observe a significant decrease in the size and density of MG acinar units. MGs appear small and irregular with periglandular fibrosis and a restricted and not well-defined lumen, and occluded by a hyper-reflective and dense meibum [45,46,47]. In addition to the instability of the tear film, another risk factor is the use of antihistamines in the long term that have anticholinergic properties due to their high affinity for the muscarinic receptor [45,46,47].

#### 2.4.4. Dry Eye and Refractive/Cataract Surgery

Laser in situ keratomileusis (LASIK) and photorefractive keratectomy (PRK) are the two most commonly performed corneal surgeries used to correct refractive errors. Negative effects on the tear film are known to occur after both procedures, despite a full achievement of the refractive result. Consequently, tear substitutes are needed in the first few postoperative months [48].

The same occurs in cataract surgery. Indeed, Li et al. have analyzed the pathogenic factors in 37 patients, and they have found that the incidence of dry eye increases dramatically after phacoemulsification [49]. The number of patients who had positive corneal and conjunctival fluorescein staining increased after surgery (16 patients; 22 eyes). The fluctuations and/or decrease of Schirmer test score, BUT, and average density of globet cells in conjunctiva followed a pattern which was in accordance with the observed symptoms of dry eye, and they were significantly worse than before cataract surgery. The ocular discomfort at 1 month was present in 14.7% of the patients; the score reached a peak at 1 month after surgery and decreased gradually after 3 months [49].

Yu and colleagues found that 95% of patients who underwent LASIK had DE symptoms during the first postoperative day, and 85% continued to experience symptoms during the first postoperative week. Over 60% of patients still had complaints of DE at the first postoperative month [50]. These symptoms improved over the course of several months, with reported rates of DE disease at 6 months ranging from 12 to 48% [51]. The incidence of DE symptoms after PRK is similar to that after LASIK except in the early postoperative period. Indeed, immediately after PRK, patients have a high level of discomfort due to the epithelium damage. Later symptoms are comparable to the post-LASIK setting. Stephenson and colleagues showed that only 20% of patients post PRK complained of DE at 6 months, and in a 12-year study [52] only 3% of patients had symptoms of DE [53].

The genesis of postrefractive DE depends on the type of technique, which causes injury to the nerve plexus leading to neurotrophic deficits. In LASIK, the nerve plexus is compromised by the execution of the corneal flap, and the nervous integrity is preserved only at the level of the hinge. Further damage is related to the extent of ablation and to the refractive correction. In PRK, injury to the nerve endings involves the entire surface ablation, and is correlated with the ablation’s depth and therefore with the amount of the refractive defect.

In cataract surgery, the damage is related to corneal and limbal incisions. The damage to the nervous plexus impairs corneal sensitivity that eventually reduces blinking, increases tear film evaporation, and alters the stimulation of tear production. [54]. The disruption of corneal innervation also lead to a loss of epitheliotropic factors, such as substance P, which have an important role for epithelial regeneration and integrity. Nerve Growth Factor (NGF) levels are reduced after LASIK or PRK, inducing a possible neurotrophic epithelial disease [55,56]. The increased evaporation or reduced tear production leads to an increase of tear osmolarity and inflammatory changes on the ocular surface. The mediators, released by damage to the nerve endings and cells after corneal photoablation, cause the degranulation of mast cells and the recall of the leukocytes, monocytes, and macrophages determining blood vessel dilation and tissue hypersensitivity [57,58].

In addition, the modification of the corneal profile alters the interaction between the upper eyelid and corneal curvature, resulting in an irregular tear distribution during blinking [59,60]. Despite that the phenomena related to the nervous trophism tend to be exhausted 6–8 months after surgery, the symptoms may persist for a longer time because of partial nerve plexus recovery or pre-existing nerve plexus pathologies. Therefore, adequate preoperative screening is required to identify and avoid refractive surgery in patients with compromised conditions.

### 2.5. Tear Film and Specific Pathologies of the Ocular Surface

#### 2.5.1. Stevens–Johnson Syndrome and Toxic Epidermal Necrolysis

Stevens–Johnson syndrome (SJS) and toxic epidermal necrolysis (TEN) are severe, immunologic dermato-bullous conditions with high morbidity and mortality that affect the skin and at least two mucous membranes. The diagnosis is made through clinical signs and a skin biopsy demonstrating full-thickness necrosis of the epidermis and keratinocyte apoptosis, with minimal involvement of the underlying dermis. SJS is defined when the denuded cutaneous surface covers less than 10% of the body surface area, and TEN is when over 30% of the body surface area is denuded. The etiology of the diseases, in addition to genetic susceptibility, is immune-mediated and can be triggered by drugs (most commonly sulfonamides, allopurinol, carbamazepine, etc.), or, less commonly, by systemic viral or mycoplasma pneumonia infection. The ocular surface represents one of the major targets in this disease, and patients may become irreversibly blind. Ocular surface inflammation can be intense, with pseudomembrane or membrane formation, symblepharon, corneal ulceration, and perforation. Acute ocular manifestations led to chronic ocular sequelae with visual impairment in at least one-third of patients. Ectropion, entropion, trichiasis, distichiasis, corneal epithelial defects, infection, neovascularization, loss of conjunctival goblet cells, meibomian gland atrophy and thickening, keratinization of the eyelid margin, and tarsal and bulbar conjunctival surfaces can be associated. Scarring in the fornices and in the lacrimal gland ducts causes severe aqueous and mucous lipid tear deficiency and xerosis. Resultant corneal blindness due to the absence of tears, eyelid malpositions, and tarsal conjunctival keratinization is the most dreaded long-term complication among SJS/TEN survivors. These changes not only cause debilitating pain in affected patients, but also threaten vision and are correlated with the development of late corneal blindness, due in part to chronic limbal stem cell dysfunction (LSCD). In general, SJS and TEN are considered T-cell mediated, type IV hypersensitivity disorders. There are elevated levels of TNF-α in the blister fluid, skin, mononuclear cells, and blood of affected patients. TNF-α activates TNF-R1, which leads to the activation of the Fas-associated death domain protein and downstream caspase pathways. Furthermore, human ocular surface epithelial cells strongly express toll-like receptor 3 (TLR3), and its ligand, polyI:C. These were found to induce various molecules, such as proinflammatory cytokines and antiviral- and allergy-related molecules, in the SJS/TEN population. To conclude, keratinocyte and epithelial cell death in SJS/TEN appears to occur by apoptosis, thus, several mechanisms are likely involved to varying degrees. Similar findings have characterized the autoimmune response in patients with chronic graft-versus-host disease (GVHD), one of the major causes of morbidity and mortality in patients undergoing allogenetic hematopoietic stem cell transplantation for hematologic malignancies. In this setting, we may find glandular dysfunction from glandular atrophy and fibrosis [61,62,63].

#### 2.5.2. Keratoconus

Keratoconus (KC), by definition, is a progressive disorder characterized by non-inflammatory corneal ectasia and stromal thinning that leads to corneal surface distortion [64,65]. The etiology of this disease remains unknown, although there is evidence of a genetic origin, and it can be associated with systemic diseases [64,65]. Eighty percent of patients suffering from KC have tired eye, irritation, and a foreign body sensation, suggesting ocular dryness [64]. In fact, while the amount of the aqueous component remains comparable to that of healthy eyes, in functional studies evaluating the tear ducts of patients with KC, BUT values tests correlate to the disease’s severity. The conical morphology of the cornea with an abnormal distribution of tears, the alteration of the quality/quantity of mucin secretion, and the reduction of GCs would lead to tear film instability [64]. Recently, the level of adenosine tetraphosphate (Ap4A) was proposed as a potential molecular biomarker for DE. Ap4A is assessed by chromatography on a strip used for the Schirmer test. Ap4A concentration in tears increases proportionally to DE severity, and patients with KC have concentrations 20 times higher than in healthy eyes [64]. The impression cytology reveals a high degree of squamous metaplasia in the conjunctival epithelium, and a marked reduction of GCs, detected in combination with confocal microscopy.

Another finding is the abnormal relationship between the cornea and the ocular surface–eyelid complex, which is also caused by the more pronounced tendency of patients with KC to rub their eyes. The morphological and functional alteration leads to an abnormal distribution of an already altered tear film. The proof is given by fluorescein and rose bengal tests; the increase of corneal accumulation of these dyes is directly related to the ocular surface damage in patients with KC [65]. Several studies report a reduction in corneal sensitivity in patients with KC, especially in those using CL. This is related to morphological and structural changes of the cornea, and to the alterations of the lacrimal function [66,67]. Moreover, histopathological and confocal microscopy-based studies demonstrated a decrease and an abnormal morphology of sub-basal and stromal nerves of the cornea in patients with KC, especially near the apex. This alteration tends to decrease, especially towards the corneal periphery [67]. The altered structure is related, in particular, to the cold thermoceptors, which are reduced in number and in a chronic excitatory state. This alteration together with tear film hyperosmolarity seems to be one of the main causes of reduced sensitivity in DE.

Furthermore, reduced levels of lactoferrin, immunoglobulin A, and lipofilina A and C together with elevated levels of serum albumin have been found in the tears of patients with KC, suggesting the presence of inflammatory processes on the ocular surface [68]. Increased levels of MMP-1, MMP-3, MMP-7, and MMP-9 have also been found in subjects with KC compared to healthy individuals [67,68]. MMPs are enzymes responsible for the degradation of the matrix of extracellular proteins, and are secreted in response to cytokines (IL-4, IL-5, IL-6, and IL-8) and growth factors (TNF-α and TNF-β).

#### 2.5.3. Pterygium

Pterygium is a common disease of the ocular surface characterized by fibrovascular tissue growth from the bulbar conjunctiva to the cornea. It may lead to chronic ocular irritation, astigmatism, tear film disorders, and decreased vision secondary to the growth of the lesion over the visual axis [69,70]. Although the exact etiology is unknown, exposure to ultraviolet (UV) radiation is believed to be the main risk factor [69,70]. Age, chronic inflammation, microtrauma, and hereditary factors can contribute to the growth of pterygium [70,71]. Studies show that the genetic trauma mediated by UV rays could alter the expression of cytokines, such as IL-6 and IL-8, in patients with pterygium [69]. IL-6 and IL-8 can induce the production of MMPs, which have been found in the advanced stage of the disease [70]. The release of IL-6, IL-8, and MMP in the tear film could lead to damage to surface and tear film instability, resulting in apoptosis of epithelial cells, GC loss, reduction of mucous secretion, and lacrimal hyperosmolarity [70,71]. Finally, it develops a vicious circle in which the tear hyperosmolarity stimulates the expression of MMPs leading to inflammation of the ocular surface [71]. A study shows that the excision of pterygium increases conjunctival GCs density and the lacrimal mucous component, improving both the osmolarity and the stability of the tear film, and consequently the BUT [72]. However, tear osmolarity tends to remain stable until the pterygium’s recurrence.

### 2.6. Up-To-Date on Biochemical Mechanisms in Dry Eye Disease

**Figure 2:**
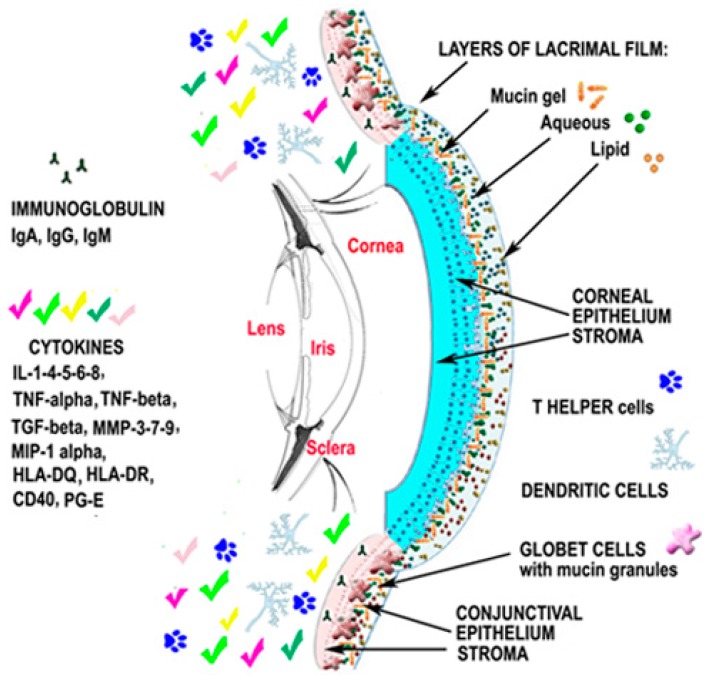
Dry eye results in the inflammatory response to diseases, infections, and damage of the ocular surface, and tear hyperosmolarity can activate a chain of events resulting in a vicious circle of inflammation of the anterior eye. Successively, the release of proinflammatory molecules secreted by several cells leads to the recruitment of immune cells as well as a disruption of the ocular surface. Interleukin-1-4-5-6-8 (IL-1-4-5-6-8); tumor necrosis factor-α (TNF-α); tumor necrosis factor-β (TNF-β); metalloproteases-3-7-9 (MMP-3-7-9); macrophage inflammatory proteins (MIP-1α); conjunctival human leukocyte antigen expression (HLA-DQ; HLA-DR); cell surface receptors (CD40 protein, CD40 ligand ICAM-1); prostaglandin-E (PG-E).

It is now established that DE is a disease caused by an inflammatory process that disrupts the normal homeostasis of the ocular surface. Thus, a common pathogenic mechanism of DE is related to the tear hyperosmolarity that can activate a chain of events resulting in a vicious circle of inflammation, which leads to further ocular surface injury (Figure 2). As a result, high levels of chemokines, macrophage inflammatory proteins (MIP-1α), cytokines (IL-1β), MMP, TNF-α, lymphocytes infiltration, and an increase of conjunctival human leukocyte antigen expression (HLA-DQ and HLA-DR), CD40 protein, CD40 ligand ICAM-1 (cell surface receptors), and apoptotic marker APO2.7 have been demonstrated in DE patients [73]. In fact, studies have confirmed the anti-inflammatory effects of CF-101, an A3 adenosine receptor (A3-AR) agonist, that binds to A3-AR and mediates the downregulation of the nuclear factor kappa-light-chain-enhancer of activated B cells-TNF-α (NF-κB-TNF-α) B-TNF-α) signaling pathway, the inhibition of cytokines secretion, and inflammatory cells apoptosis [73]. It has been suggested that the improvement of several pathological signs and symptoms in subjects with DE, such as Schirmer test and BUT scores, might be due to a reduction in ocular surface inflammation following direct interaction between CF101 and its receptors on inflammatory cells [73].

Furthermore, other recent studies have highlighted the essential role of inflammatory mediators. Since Omega 3 (ω3) and Omega 6 (ω6) are considered polyunsaturated fatty acids (PUFAs), they are defined as “essential” and they cannot be synthesized by the human body; ω3 and ω6 derive from α-linolenic acid and linoleic acid, respectively, and they are precursors of eicosanoids with potential anti-inflammatory (ω3 group) and proinflammatory effects (ω6 group). An equilibrate balance between ω3 and ω6 is necessary to avoid the prevalence of an ω6 proinflammatory effect. In particular, proresolving lipid mediators derived from ω-3 precursors, such as docosahexaenoic acid (DHA) and eicopentaenoic acid (EPA), control epithelial wound healing, inflammatory cell migration, and nerve regeneration [74,75]. The prostaglandins, belonging to a class of inflammatory lipid mediators called eicosanoids, are derived from the oxygenation of arachidonic acid (AA) and ω-6 that is enzymatically released from the cell membranes of activated cells in response to environmental stress. The release of AA and the subsequent generation of eicosanoid lipid mediators is responsible for triggering the acute inflammatory response to corneal injury [73]. However, its resolution is mediated by proresolving lipid mediators derived from the ω-3 PUFA precursors DHA and EPA. Consequently, it is hypothesized that DE is a metabolic disorder characterized by an imbalance of ω-3 and ω-6 PUFA leading to the underproduction of proresolving lipid mediators.

**Figure 3:**
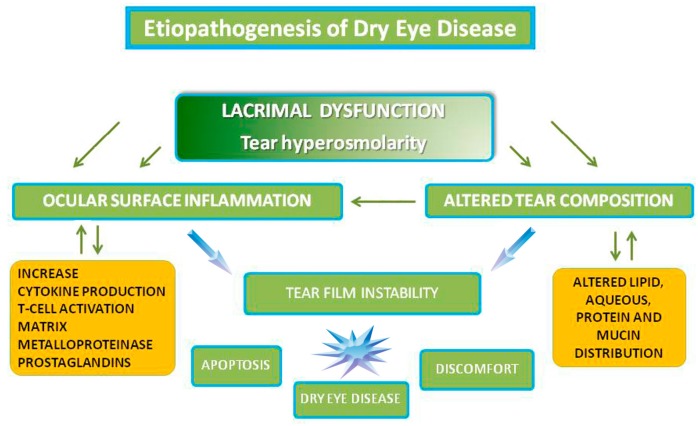
Etiopathogenesis of Dry Eye Disease.

Corneal epithelial cells and resident regulatory polymorphonuclear (PNMs) leukocytes in the corneal limbus and lacrimal gland highly express 15-lipoxygenase (15-LOX), a key enzyme for the generationg and release of specialized proresolving mediators, which are critical for the control of the immunity of the ocular surface. Endogenous lipoxin A4 promotes corneal epithelial wound healing [76], inhibits pathological angiogenesis and proinflammatory cytokine expression [77,78], and controls effector T-cell activation [79]. Neuroprotectin D1 is implicated in epithelial cell survival [80], recovery from oxidative stress, and wound healing [81]; it has also been shown to promote corneal nerve regeneration and corneal sensitivity restoration [81,82]. Resolvin E1 exerts proresolving effects directly through G-protein-coupled receptors [83] and indirectly through negative feedback on cyclooxygenase-2 (COX-2) expression, which plays an important role in the synthesis of prostaglandins from arachidonic acid [84]. It has been shown that these mediators are essential for the homeostasis of the tear film, the survival of GCs, and the secretion in response to stress. This underlines the biological significance of ω-3 proresolving lipid mediators in the tear film, and supports the hypothesis that DE has a metabolic basis. The nutritional or metabolic deficiency of DHA and EPA may lead to an underproduction of lipid mediators in tears. A higher ratio of ω-6 lipid mediators of ω-3 induces the inflammation of the ocular surface. Recent studies and a meta-analysis of randomized controlled trials support the supplement of ω-3 fatty acids for DE syndrome; oral administration of ω-3 improves Schirmer Test and BUT scores (Figure 3) [81,82,83,84].

### 2.7. Therapeutical Pragmatic Approach

The purpose of recent research was to promote ocular surface health by reducing the inflammation and stabilizing the tear film. Although several therapeutic approaches for DE are available, therapeutic strategies, such as the use of artificial eye lubricants, focus on the treatment of symptoms guaranteeing just temporary relief to the patient without having an effect on the implicit causes of DE [12]. The DEWS report suggests that a clinician choose specific treatments based on the severity of the disease according to patients’ clinical history and experience [2]. Nowadays, treatments of DE include the use of lubricating eye drops, unguents, dietary supplements associated with eyelid cleaning, and in severe cases with punctual occlusion and topical and oral non-steroidal anti-inflammatory (NSAID) and corticosteroid drugs.

#### 2.7.1. Topical Nonsteroidal Anti-Inflammatory (NSIAD)

NSAID and steroids are usually evaluated for SS even if a prolonged use of steroids may be associated with severe side effects such as the onset of cataract, infections, and a high level of intraocular pressure [12]. Currently, there are few NSAIDs that do not seem to cause toxic effects on the cornea; however, they could lead to DE by reducing eye corneal sensitivity [85,86].

Pranoprofen and bromfenac sodium ophthalmic solution are NSAIDs with an anti-inflammatory effect due to the inhibition of epoxidase and synthesis of arachidonic acid (AA). These drugs improved signs and symptoms, and reduced conjunctival human leukocyte antigen II (HLADR) expression in patients, also in cases where patients were unresponsive to lubricant monotherapy [85,86].

Thymosin β4 is a naturally occurring 43 amino acid peptide found in high concentrations in most tissues, cells, blood plasma, and ocular surface fluid. Thymosin β4 possesses both wound-healing and anti-inflammatory properties [12,87]. This drug has been shown to have a positive effect on epithelial migration and healing, as well as an anti-inflammatory effect through a number of different pathways in vitro, in patients with diabetic neurotrophic corneal defects, and in severe DE as well as graft-versus-host disease (GVHD), SJS, and TEN [61,63,87].

#### 2.7.2. New Topical Natural Substance

Proteoglycan 4 (PRG4), also called lubricin, is an amphiphilic glycoprotein which plays a critical role as a boundary lubricant in several sites throughout the body [88,89].

Some studies have discovered the presence of lubricin mRNA in a number of exocrine and reproductive tissues. This superficial zone protein, containing a 1404 amino acid core, is produced by ocular surface epithelium, and acts in protecting the cornea and conjunctiva against shear forces generated during an eyelid blink [88,89].

A deficit of this glycoprotein, a natural boundary lubricant, promotes shear stress on the ocular surface and increases damage. Conversely, the exogenous application of lubricin significantly reduces the friction between the cornea and conjunctiva. In a recent clinical trial, the researchers stated that the efficacy and safety of recombinant human lubricin compared to a 0.18% hyaluronic acid (HA) eye drop in 39 subjects with moderate DE [89,90].

#### 2.7.3. Secretagogues

Different topical pharmacologic agents may potentially increase both aqueous and mucin secretion. Some preclinical studies have demonstrated the efficacy of Nerve Growth Factor in DE. In fact, NGF supports the lacrimal function, stimulating the secretion of glycoconjugates from conjunctival cells and promoting corneal homeostasis. NGF’s trophic function is related to tyrosine kinase receptor (TrkA) and p75 neurotrophin receptor (p75 NTR) stimulation. The presence of both TrkA and NGF on the ocular surface highlights their role in promoting ocular homeostasis. By binding TrkA, NGF stimulates the release of mucin by the GCs of the conjunctiva, stabilizing the tear film [91].

MIM-D3 is a small protein-like chain designed to mimic NGF; it activates the TrkA receptor, but is not able to bind the p75NTR receptor. In a Phase II clinical trial, Meerovitch et al. demonstrated a considerable improvement in the signs and symptoms of DE, with both 1% and 5% MIM-D3 ophthalmic solutions. The 1% solution was related to the best result in fluorescein and lissamine green corneal and conjunctival staining, respectively [91].

More recently, 3% diquafosol tetrasodium, a P2Y2 purinergic receptor agonist, has been launched as an ophthalmic solution in Asian countries. It acts through a novel pathway by activating P2Y2 receptors of the ocular surface, and stimulating the quantity and quality of tear fluid secretion, as determined by optical coherence tomography (OCT) and meniscometry in DE [92].

Diquafosol also stimulates the secretion of sialic acid, which is a mucin-like substance, in tears in healthy subjects after a single dosing. Therefore, clinical trials demonstrated a good safety profile with clinical improvement of the ocular surface in severe DE patients [92,93].

#### 2.7.4. Topical Immunomodulators

Cyclosporine A (0.05–2%), a lipophilic cyclic polypeptide, is the first topical immunomodulator in ophthalmic emulsion approved by the U.S. FDA in 2002 and by the European Union in 2015. Cyclosporine A acts as an immunosuppressive agent when administered systemically. It has been used topically for blepharitis, dry eye, keratoconjunctivitis, post LASIK, GVHD, SJS, and TEN [94,95,96,97].

It has been shown to inhibit the production and/or release of proinflammatory cytokines and to upregulate the release of anti-inflammatory cytokines [94,96]. Moreover, cyclosporine A inhibits the apoptosis and the amount of Fas-ligand expression in the infiltrating lymphocytes of human conjunctival epithelial cells [96,97]. The U.S. FDA approved on 11 July 2016 the first medication of a new class of drugs for the treatment of DE. Lifitegrast, in topical administration, is a novel integrin antagonist which inhibits proinflammatory T-cell activity by T-cell adhesion to intercellular adhesion molecule-1 (ICAM-1) [94,98].

#### 2.7.5. Systemic Therapy

Some studies have investigated the effects of dietary integration with PUFAs as a possible substitutive therapy in the management of DE. The authors concluded that a metabolic deficiency of ω3 tear film lipids can cause an inflammatory impact on the ocular surface, and this could lead to chronic inflammation in DE subjects. Further large-scale, multicenter studies are necessary to establish the guidelines for the administration and dosing of PUFAs [12]. Recently, new biologic agents targeting B-cells, such as rituximab, belimumab, and epratuzumab, have shown promising results in the treatment of autoimmune and progressive conditions such as SS. Achievements with rituximab, monoclonal antibody directed against CD20, and other B-cell-depleting therapies, such as interferon-α (INF-α) and anti-B-cell-activating factor (BAFF), have shown potential benefit even in severe DE [99,100,101,102]; thus, further studies are needed to validate the findings in these patients.

## 3. Conclusions

DE syndrome consists of a wide spectrum of disorders with different causes. Nowadays, artificial tears that guarantee a transient improvement of DE patients’ symptoms represent the first and most frequently used therapy for a distressing condition that significantly impacts quality of life. However, untreated inflammation can lead to severe and permanent complications. The aim of new medications is to affect the various pathogenetic factors involved in the onset of DE. Nevertheless, new clinical trials have demonstrated the effectiveness of stem cell transplantation-based treatments from human embryonic stem cells (hESCs), which induce pluripotent stem cell (iPSC) growth [103,104]. Stem cells hold promise for treating severe eye diseases, as the eye is an ideal organ for such an approach, due to its relative immunological privilege, surgical accessibility, and the fact that it is a self-contained system [87,103,104].

A multidisciplinary approach based on both topical and systemic therapies should be considered for the clinical management of DE in order to obtain sustained relief for patients. Further studies are needed to investigate the long-term effects of these new therapies planned for eventual treatment of this chronic multivariate disease.

## Figures and Tables

**Table 1:** Dry eye symptoms and diagnostic tests.

**Common Dry Eye Symptoms**	
Irritation	Burning or stinging sensation
Dryness or grittiness	Foreign body sensation
Itching and Redness	Tearing
Fluctuation of vision	Contact lens intolerance
Increased blinking frequency	Blurry vision
Photophobia	Mucous discharge
**Dry Eye Diagnostic Tests**	
Tear Break Up Time (BUT)	Tear film meniscus height
Ocular surface staining with fluorescein, lissamine green, and rose bengal dye	Schirmer test I and II
Other tests: tear film osmolarity, lactoferrin, lysozyme immunoglobulin, and albumin dosage, and impression cytology.	

**Table 2:** Dry eye (DE) classification according to pathogenetic analysis in ocular surface disorders.

Meibomian Glands Dysfunction	Principal Mixed Forms	Other Major Mixed Forms	Aqueous Deficits	Ocular Surface Diseases
Primary or DE blepharitis syndrome (DEBS). Secondary to skin diseases such as rosacea, psoriasis, lupus, ichthyosis, rheumatoid arthritis, etc.	Oxidative stress, aging and/or associated factors. Corneal hypoesthesia. Systemic/topical medications, conjunctivochalasis, abnormal eyelid position, etc.	Hormonal changes, menopause, pregnancy. Allergic diseases, bacterial or viral conjunctivitis, use of contact lenses or computer, ocular surface trauma or tumor or surgery, environmental factors, etc.	Autoimmune pathologies: Sjögren’s syndrome dry eye (SS). Non-Sjögren’s syndrome dry eye (non-SSDE)	Stevens Johnson syndrome (SJS), toxic epidermal necrolysis (TEN), graft-versus-host disease (GVHD). Neurotrophic deficiencies, Keratoconus (KC), pinguecula, pterygium, corneal dystrophies, etc.
